# Nanochips of Tantalum Oxide Nanodots as artificial-microenvironments for monitoring Ovarian cancer progressiveness

**DOI:** 10.1038/srep31998

**Published:** 2016-08-18

**Authors:** Udesh Dhawan, Ssu-Meng Wang, Ying Hao Chu, Guewha S. Huang, Yan Ren Lin, Yao Ching Hung, Wen Liang Chen

**Affiliations:** 1 Department of Materials Science and Engineering, National Chiao Tung University, Hsinchu 1001 University Road, Hsinchu, Taiwan, 300, ROC; 2 Hokan Life Technology, F2, 793 Fu-Ke Road, Taichung, Taiwan, 402, ROC; 3 Department of Emergency Medicine, Changhua Christian Hospital, Changhua, Taiwan; 4 School of Medicine, Kaohsiung Medical University, Kaohsiung, Taiwan; 5 School of Medicine, Chung Shan Medical University, Taichung, Taiwan, 402, ROC; 6 Department of Obstetrics and Gynecology, China Medical University Hospital, Taichung, Taiwan, 404, ROC; 7 Department of Biological Science and Technology, National Chiao Tung University, Hsinchu, 1001 University Road, Hsinchu, Taiwan 300, ROC

## Abstract

Nanotopography modulates cell characteristics and cell behavior. Nanotopological cues can be exploited to investigate the *in-vivo* modulation of cell characteristics by the cellular microenvironment. However, the studies explaining the modulation of tumor cell characteristics and identifying the transition step in cancer progressiveness are scarce. Here, we engineered nanochips comprising of Tantalum oxide nanodot arrays of 10, 50, 100 and 200 nm as artificial microenvironments to study the modulation of cancer cell behavior. Clinical samples of different types of Ovarian cancer at different stages were obtained, primary cultures were established and then seeded on different nanochips. Immunofluorescence (IF) was performed to compare the morphologies and cell characteristics. Indices corresponding to cell characteristics were defined. A statistical comparison of the cell characteristics in response to the nanochips was performed. The cells displayed differential growth parameters. Morphology, Viability, focal adhesions, microfilament bundles and cell area were modulated by the nanochips which can be used as a measure to study the cancer progressiveness. The ease of fabrication of nanochips ensures mass-production. The ability of the nanochips to act as artificial microenvironments and modulate cell behavior may lead to further prospects in the markerless monitoring of the progressiveness and ultimately, improving the prognosis of Ovarian cancer.

Nanotopography can regulate cellular behavior. Topographies such as nanodots^1^,^2^,^3^,^4^,^5^, nano-islands^6^, nano-concave^7^, nano-diamond, nano-groove^8^,^9^,^10^,^11^, nano-tube^12^, nano-ridge^13^,^14^, nano-pore^15^ which show high biocompatibilities have been seen to control the cell physiology, cell growth, migration and cell adhesion. Several 2D surfaces made us from materials such as Titanium dioxide^16^,^17^,^18^ (TiO_2_), as well as certain 3D structures^19^ and polymers^20^ have recently been discovered to possess the capability to modulate cellular behavior. Osteoblasts have been seen to change morphology in response to nanopography^21^,^22^. Nanodot arrays have also been seen to modulate the cell characteristics such as cytoskeletal organization, cell viability, focal adhesions, microfilament bundle density, apoptosis in the Ovarian Cancer cell lines TOV-112D, TOV-21G, and cervical cancer cell line C33A^23^. Tantalum oxide nanodot arrays in specific, have shown a tremendous potential to guide not only the cellular behavior but also modulate the genetic constitution of the cells^1^,^4^,^5^,^24^,^25^. All of these studies collectively demonstrate that nanotopography can control and modulate cellular behavior and parameters *in-vitro*^26^. However, *in-vivo*, cellular microenvironment is responsible for controlling the cell behavior^27^ and also acts a transition-inducing factor during cancer progressiveness^28^,^29^,^30^,^31^. Tumor cells encounter specific changes in their surrounding microenvironment which assist them in evolving from less to a more progressive form^32^.

The cellular microenvironment components, collagen microfibrils in the Extra cellular matrix (ECM), for instance, comprises of topographical features^33^,^34^,^35^ which lie from 20 to 200 nm. These topographical features continuously interact with the cells to modulate their behavior^36^,^37^,^38^,^39^. In extension, the cells too possess features such as focal adhesions, cilia, filopodia which lie in the nano-range. The cells and the cellular microenvironment interact and modulate each other on a nanoscale due to this similarity in their features. Therefore, engineering topographies in the nanoscale can mark as one possible way to understand these interactions where the nanotopographies mechanically behave in a manner similar to the cellular microenvironment components.

Most, but not all of the cancers have specific markers which can be studied to diagnose the cancer progressiveness. Ovarian cancer is the leading cause of death among gynecological cancers in females due to the lack of any specific marker to diagnose the disease progression. Most patients are present with the advanced disease and have a poor prognosis with present therapies. Even though the early stage survival rate is over 90%, however, only 20% of the cases are caught in the early stages which brings the 5-year survival rate to a mere 11% after having been detected in the advanced stage (III/IV). The histologic grade of a tumor measures how abnormal or malignant its cells look under the microscope. Even though a plethora of studies have been done to study the regulation of normal cell behavior by nano-materials^1^,^5^,^23^, however, none of the studies have aimed to address the challenge of monitoring the Ovarian cancer progressiveness by studying the control of cell characteristics in response to nanotopography. Based on the findings of our previous studies, it may be possible to utilize nanotopographies engineered from Tantalum oxide as a way to study the cancer progressiveness^23^. It is, therefore, an immediate need for the scientific community to devise a platform which can be used to study and monitor the Ovarian cancer invasiveness as a measure of modulation of cell behavior and characteristics by nanomaterials.

The present study is based on the hypothesis that Tantalum Oxide nanodot arrays of different sizes can act as artificial microenvironments to the Ovarian cancer cells, modulate the cellular characteristics and serve as a factor to induce a transition in cell characteristics to study the invasiveness of the cancer sample. Nanochips consisting of nanodot arrays of size varying from 10 nm to 200 nm was fabricated to act as artificial microenvironments. Clinical Ovarian cancer samples at different stages of Serous, Mucinous, and Clear cell carcinoma were obtained and primary cultures were established. Primary cultures were then seeded on the nanochips which consisted of a variety of nanodot sizes and cell characteristics were evaluated. The correlation of nanochip’s derived growth indices to clinical-pathological disease progression was established. Investigation of cell-nanomaterial interaction to provide a theoretical basis for the modulation of cell characteristics was also performed. The nanochips were also optimized to improve the integration, convenience, economy and enhanced accuracy. The primary goal of this research is to develop a platform, to assist in the clinical-pathological monitoring of Ovarian cancer progressiveness. According to our results, these nanochips are capable of distinguishing between different stages of different types of Ovarian cancer. These nanochips can be unified to serve as an ideal platform for studying cancer progression and improving Ovarian cancer prognosis.

## Results

### Fabrication of nanochips of Tantalum oxide nanodots arrays

**Figure 1:**
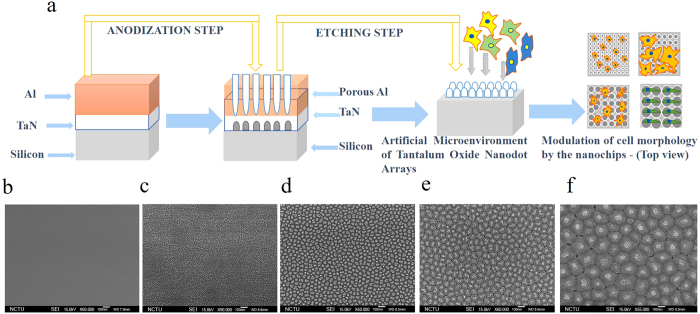
Scanning electron microscopy (SEM) of Tantalum oxide nanodots. (**a**) Schematic representation of fabrication of nanochips comprising of Tantalum oxide nanodots as artificial microenvironment (**b–f**): SEM of Tantalum oxide nanodots; Flat, 10, 50, 100 and 200 nm, respectively. Scale bar = 100 nm.

Nanochips, comprising of Tantalum oxide Nanodot arrays, which served as artificial microenvironments for this study were fabricated by anodic aluminum oxide (AAO) processing on the aluminum-tantalum-coated wafer (Fig. 1a). Tantalum oxide nanodots array with 10, 50, 100, and 200 nm dot diameters were fabricated on a silicon wafer. The nanodot diameters 12 ± 2.8, 50.35 ± 3.2, 99.4 ± 6.3, and 206.7 ± 6.5 nm (Fig. 1b) were examined with scanning electron microscopy (JEOL JSM-6500 TFE-SEM). Dimensions of nanodots were well controlled and highly defined.

### Morphology and focal adhesion analysis by Immunostaining Cytoskeleton and Vinculin

**Figure 2:**
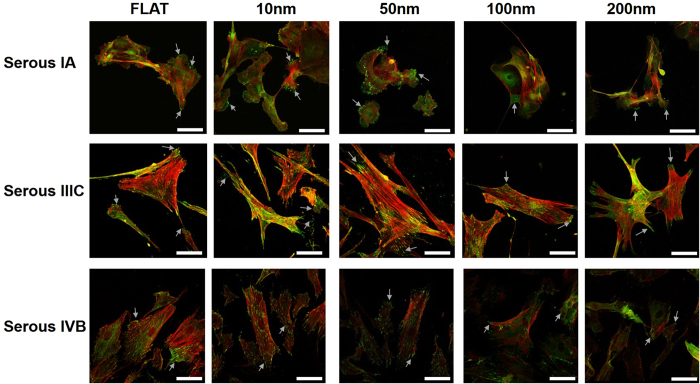
Immunofluorescent images of Cytoskeletal arrangement and vinculin distribution in Serous type Ovarian cancer cells in different stages (IA, IIIC and IVB). Cells were seeded on different nanodot arrays (Flat, 10, 50, 100 and 200 nm) and harvested after 3 days. Cytoskeleton arrangement was examined using Phalloidin (Red stain) and Vinculin distribution was examined using anti-Vinculin antibodies (Green dots/green-stained area). Arrows represent the focal adhesions points of cells to the Nanochips. All scale bars = 75 μm.

**Figure 3:**
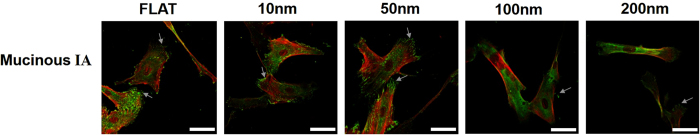
Immunofluorescent images of Mucinous type Ovarian cancer cells in stage IA. Cells were seeded on different nanodot arrays (Flat, 10, 50, 100 and 200 nm) and harvested after 3 days. Cytoskeleton arrangement was examined using Phalloidin (Red stain) and Vinculin distribution was examined using anti-Vinculin antibodies (Green dots/green-stained area). Arrows represent the focal adhesions points of cells to the Nanochips. All scale bars = 75 μm.

**Figure 4:**
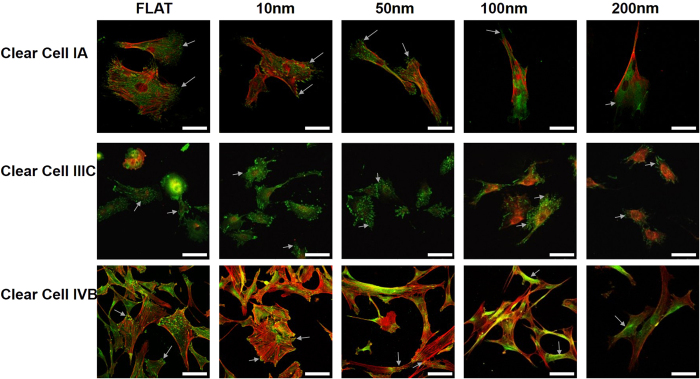
Immunofluorescent images of Clear Cell type Ovarian cancer cells in different stages (IA, IIIC and IVB). Cells were seeded on different nanodot arrays (Flat, 10, 50, 100 and 200 nm) and harvested after 3 days. Cytoskeleton arrangement was examined using Phalloidin (Red stain) and Vinculin distribution was examined using anti-Vinculin antibodies (Green dots/green-stained area). Arrows represent the focal adhesions points of cells to the Nanochips. All scale bars = 75 μm.

Nanochips, comprising of Tantalum oxide nanodot arrays, which served as artificial microenvironments for the growth of cancer cells modulated the morphology of Ovarian cancer cells in different stages. The modulation in the nanochip size served as a transition step in the modulation of morphology and the number of focal adhesions of the cells on the nanochips (Figs 2, 3 and 4). The expression of vinculin i.e. the number of focal adhesions (green dots) was also found to be controlled by nanochip size. Cells in stage IA of Serous Ovarian cancer exhibited a flat morphology on 10 nm Nanochips. No significant difference in the cell morphology was observed in cells on flat and 10 nm nanochips. A round morphology on 50 and 100 nm nanochips (Fig. 2) was seen. However, an elongated morphology was observed in cells on the 200 nm nanochips which indicates a morphology transition. In stage IIIC, an extended morphology was observed in cells on flat surfaces. However, cells on the 10 and 50 nm nanochips displayed a distinct spindle-shaped morphology in contrast to a flat and extended morphology on 100 and 200 nm nanochips (Fig. 2). In stage IVB, flat cell morphology on control surfaces was exhibited, however, cells on 10 and 50 nm nanochips exhibited an extended morphology which transitioned to a shrunken morphology on 100 and 200 nm nanochips. The percentage of focal adhesions decreased dramatically with the increase in the size of the nanochips for all stages (Fig. 2). Cells on 10 nm nanochips exhibited the maximum number of focal adhesions while the cells on 200 nm nanochips had almost negligible focal adhesions (Fig. 2).

For the Mucinous type of Ovarian cancer, in stage IA, the cytoskeletal organization of cells on Flat, 10 and 50 nm nanochips exhibited a flat morphology, however, on the 100 and 200 nm nanochips, cells displayed an elongated morphology (Fig. 3). Cells on all nanochips exhibited a trend in morphology transition similar to the Serous type of Ovarian cancer cells (Figs 2 and 3). The number of focal adhesions gradually decreased as the array size increased. Cells on the 10 nm nanochips displayed a maximum number of focal adhesions while cells on 200 nm had the least (Fig. 3).

For Clear cell type in stage IA, a well extended morphology was seen on flat surfaces, however, the cells on 10 nm nanochips showed a flat and extended morphology with a large cell area. However, with an increase in the nanochips size, cells started adopting an elongated or a spindle-like morphology (Fig. 4). In this case, the change in the size of the nanochips from 10 to 50 nm controlled the cell morphology and marked the transition step. For stage IIIC, cells on all nanochips (10, 50, 100, 200 nm) and flat surfaces exhibited a round shrunken morphology and no significant transition in the morphology in response to the nanochips was observed. However, distinct differences between the morphologies of cells in stage IA and IIIC were observed for nanochips which indicated the control of cell morphology. For stage IVB, cells on flat surfaces and 10 nm nanochips displayed a well-extended morphology with large cell area. However, the change in the nanochips size from 10 to 50 nm marked a transition step where cells on 50 nm nanochips had an elongated morphology which transformed to spindle-shaped cells on 100 and 200 nm nanochips (Fig. 4). The vinculin expression was similar to what was observed in Serous and Mucinous types of Ovarian cancer cells. Cells on 10 nm nanochips had the maximum focal adhesions with the nano-surface whereas cells on 200 nm showed the least. The change in the size of nanochips also caused a transition in vinculin expression. In stage IA, vinculin was expressed both in the cytoplasm and as focal adhesion in the cells on 50 nm nanochips. However, on 100 and 200 nm nanochips, vinculin was only expressed in the cytoplasm but not as focal adhesions. This indicates that a change in the array size of the nanochips can act as a measure to modulate vinculin expression.

In summary, the nanochips comprising of nanodot arrays successfully distinguished between different stages of cancer (Figs 2, 3 and 4). The change in the nanochip size from 10 to 50 nm marked as the transition in the morphologies of cells in different stages of the cancer cells (Fig. 4). In addition, distinct differences between the morphologies of cells in different stages and the consistent decrease in the number of focal adhesions could be seen which were regulated by the nanochip size.

### Measurement and comparison of Cell Indices of Serous type of Ovarian Cancer in different stages

**Figure 5:**
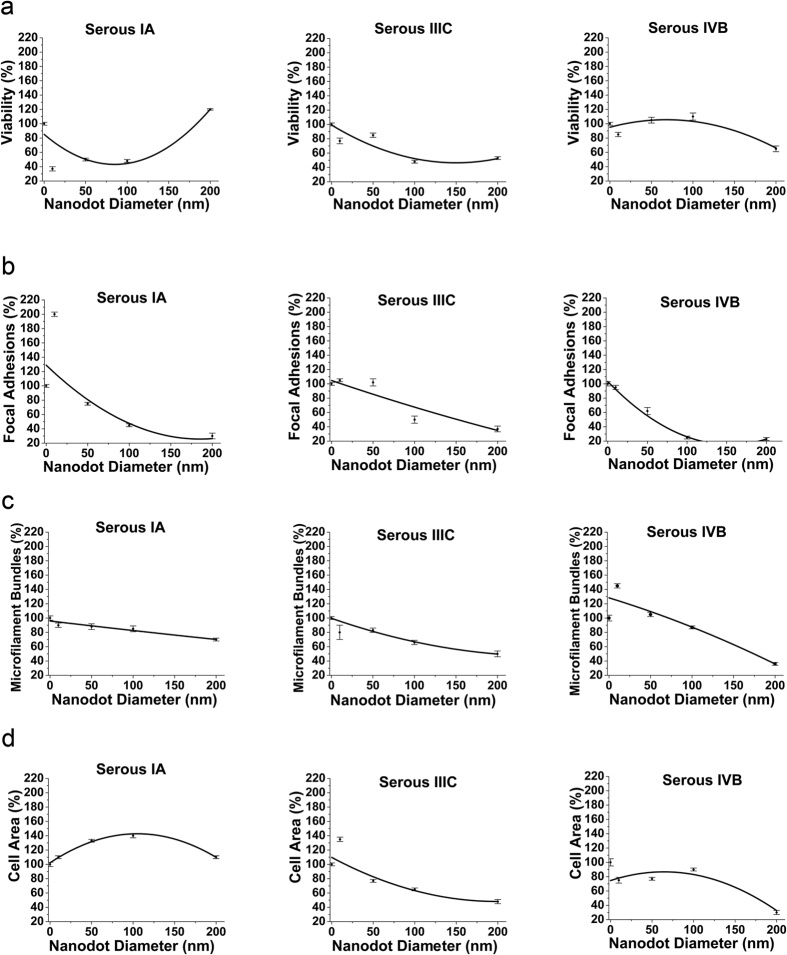
(**a**) Viability (expressed as percentage) of Serous type of Ovarian cancer in different stages (IA, IIIC, IVB) versus Nanodot diameter. (**b**) Focal adhesions of Serous type of Ovarian cancer in different stages versus Nanodot diameter. (**c**) Microfilament bundles of Serous type of Ovarian cancer in different stages versus Nanodot diameter and (**d**) Cell area of Serous type of Ovarian cancer in different stages versus Nanodot diameter. Cells were harvested after 3 days and cell characteristics were examined. Data are normalized with respect to control (flat) surfaces. The mean ± SD from 3 separate experiments is shown.

Serous is the most common epithelial Ovarian cancer. It accounts for two-thirds of the Ovarian cancer patients. Significant differences in the cell indices of different staged cells were observed. In the early stage (IA), a significant decrease of 62% was seen in cells on 10 nm nanochips compared to flat (control) surfaces. There was a steady increase in the percentage of viable cells on the 10 to 100 nm nanochips (Fig. 5a). However, an abrupt increase in the viability was observed on the nanochip greater than 100 nm. The nanochips of 100 nm marked the transition in the percentage of viable cells. Viability was maintained at 120% for cells seeded on 200 nm nanochips, as compared to control surfaces (Fig. 5a). For cells in stage IIIC, the percentage of viable cells decreased significantly. A small decrease was seen in cells on 10 nm Nanochips compared to flat surfaces. The 100 and 200 nm nanochips had the same percentage of viable cells as compared to the control (flat). Viability was maintained at 55% for cells on 200 nm nanochips. For cells in the advanced stage (IVB), the percentage of viable cells increased consistently with the increase in the nanochips size, however, as the nanochips size increased 100 nm, the percentage of viable cells dropped to 60% as compared to the control (Fig. 5a). Transition in the cell index was observed as the nanochips became greater than 100 nm. In Summary, significant differences in the viability of cells in different stages of serous type Ovarian cancer cells was observed. Percentage of viable cells on different nanochips displayed different trends in the early and advanced stage (Fig. 5a).

The percentage of focal adhesions followed a consistent trend (decrement) in all stages. In the early stage (IA), cells seeded on 10 nm nanochips displayed 200% focal adhesions as compared to the control surface (Fig. 5b). However, the percentage of focal adhesions decreased dramatically with the increase in the size of the nanochips above 10 nm. The focal adhesions dropped to 70% for cells on 50 and 30% for cells on 200 nm nanochips. For the advanced stage (IVB), the percentage of focal adhesions followed the same trend (decrement), however, the percentage of focal adhesions of cells on all nanochips was lower than in the early stage (IA). Lowest percentage of focal adhesions was 10% for cells on 200 nm nanochips (Fig. 5b). In Summary, the nanochips induced the transition in the dramatic decrease in the percentage of focal adhesions.

The percentage of microfilament bundles (MFB) followed the same trend (consistent decrement) as the number of focal adhesions, however, the transition step was seen to be induced by different nanochip size (Fig. 5c). Cells on 200 nm nanochips displayed the least percentage of focal adhesions for all stages, however, the decrease in the percentage of microfilament bundles was more drastic in the advanced stage (IVB) than in the early stage (IA). In addition, in the early stage (IA), nanochips greater than 100 nm induced the transition in the decrement of MFB, however in the advanced stage (IIIC, IVB), the transition was induced by size greater than 10 nm (Fig. 5c). In summary, nanochips greater than 100 nm induced the transition in the early stage while, in the late stage, nanochips greater than 10 nm served as the transition step.

Cells in different stages displayed different morphologies (Fig. 2) and hence different cell area (Fig. 5d). In the early stage, the area of any cell increased until the nanochips size reached 100 nm and then decreased as the size became greater than 100 nm (Fig. 5d). In brief, nanochips greater than 100 nm induced a significant morphology transition (Fig. 2) and hence the cell area (Fig. 5d). Cells on 200 nm nanochips displayed the least cell area as was complemented by the elongated morphology (Fig. 2). Cells in the advanced stage (IVB) displayed a well-extended morphology (Fig. 2) and hence a greater cell area for cells seeded on the 10 nm nanochips (Fig. 5d). However, as the nanochips size increased more than 100 nm, cells displayed a shrunken morphology (Fig. 2) which complemented the reduced cell area (30%). In brief, nanochips greater than 100 nm served as the transition step of morphology/cell area modulation.

### Measurement and comparison of Cell Indices of Mucinous type of Ovarian Cancer in different stages

**Figure 6:**
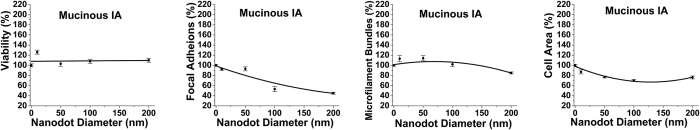
Viability, Focal adhesions, Microfilament bundles and cell area of the Mucinous type of Ovarian cancer (expressed as percentage) in stage IA. Cells were harvested after 3 days and cell characteristics were examined. Data are normalized with respect to control (flat) surfaces. The mean ± SD from 3 separate experiments is shown.

Mucinous type of Ovarian cancer is among the hardest to be interpreted and are often larger than the Serous type of Ovarian cancers. In stage IA, a small increase on the cell area was seen in cells on 10 nm nanochips compared to flat surfaces. Mucinous type Ovarian cancer cells in the early stage did not show any significant difference in viability for all nanochips (Fig. 6). However, the percentage of viable cells on 200 nm nanochips was consistent between the serous type Ovarian cancer (Fig. 5a) (stage IA) and Mucinous type of Ovarian cancer (Fig. 6) (110%, stage IA). In addition, viability dropped as the nanodot diameter on the nanochips became greater than 10 nm which marked the transition step in the control of viability and then remained consistent henceforth (Fig. 6).

The percentage of focal adhesion of mucinous type Ovarian cancer cells in the early stage (IA) (Fig. 6) onto the nanochips was consistent with the early stage (IA) Serous cells (Fig. 5b). 10 and 50 nm displayed the similar percentage of focal adhesions. However, a transition in the control of focal adhesions by the nanochips was observed as the nanochips size became greater than 50 nm (Fig. 6). A dramatic decrease in the focal adhesions was observed. Cells on 200 nm nanochips displayed the least percentage of focal adhesions.

The percentage of MFB in the early stage (IA) mucinous cells increased on 10 nm nanochips as compared to flat surfaces, remained consistent on 50 nm nanochips but showed a decrement in their percentage as the nanochips size became greater than 50 nm (Fig. 6). The decrease was consistent on 200 nm nanochips as well. In addition, the trend in the decrement of MFB was consistent between the early stage of Serous (Fig. 5c) as well as the Mucinous type of Ovarian cancer (Fig. 6).

A small yet consistent decrease in the cell area was observed till the nanochips reached 100 nm (Fig. 6). However, a small increase in the cell area was observed as the nanochips became greater than 100 nm. Interestingly, cells in early stage (IA) of Serous (Fig. 2) as well as Mucinous type (Fig. 3), displayed an elongated morphology on 200 nm nanochips. In addition, even though the cell area was found to be same between cells on 10 and 200 nm (Fig. 6), a distinct difference in the morphology was observed (Fig. 3).

### Measurement and comparison of Cell Indices of Clear-Cell type of Ovarian Cancer in different stages

**Figure 7:**
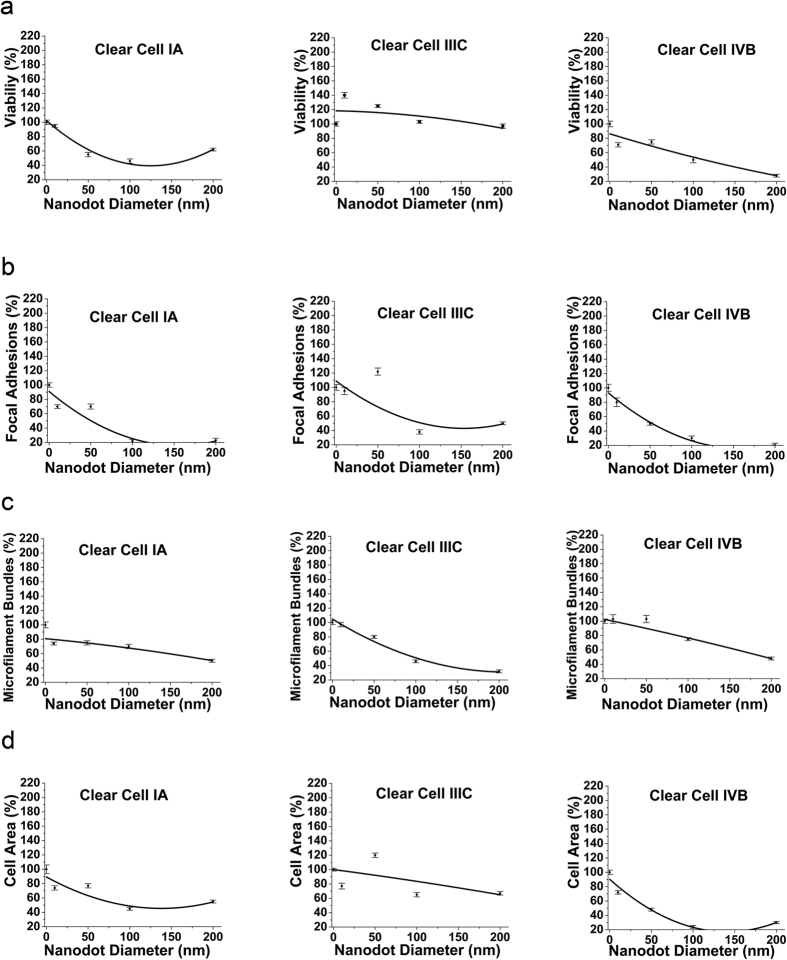
(**a**) Viability of clear cell type of Ovarian cancer in different stages (IA, IIIC, IVB) versus Nanodot diameter. (**b**) Focal adhesions of clear type of Ovarian cancer in different stages versus Nanodot diameter. (**c**) Microfilament bundles of clear cell type of Ovarian cancer in different stages versus Nanodot diameter and (**d**) Cell area of clear cell type of Ovarian cancer in different stages versus Nanodot diameter. Cells were harvested after 3 days and cell characteristics were examined. Data are normalized with respect to control (flat) surfaces. The mean ± SD from 3 separate experiments is shown.

Clear cell Ovarian carcinoma is relatively rare than Serous and Mucinous type and accounts for <5% of the cases. However, they depict the poorest prognosis as compared to all other types of Ovarian cancers. In this study, cells in the early stage (IA) displayed a decrease in the viability till the nanochips size reached 100 nm (Fig. 7a). However, as the nanochip size became more than 100 nm, an increase in the viability was observed in cells seeded on 200 nm nanochips. This finding was in agreement with the viability trend seen in early stages of Serous (Fig. 5a) and Mucinous (Fig. 6). However, the transition step in the modulation of viability was different. In the advanced stage (IVB), viability on 10 nm Nanochips was smaller as compared to flat surfaces. Interestingly, viability first increased as the nanochip size became greater than 10 nm but showed a decrement for nanochips larger than 50 nm. In this case, 50 nm was seen as the transition inducing size (Fig. 7a). Interestingly, the decrease in the viability for nanochips greater than 100 nm was consistent with the advanced stage (IVB) of Serous (Fig. 5a) type of Ovarian cancer.

The Percentage of focal adhesions on 10 nm were less as compared to on flat nanosurfaces. In the early stages (IA), the percentage of focal adhesions initially increased as the nanochips became greater than 10 nm but showed a consistent decrement as the nanochip size became greater than 50 nm (Fig. 7b). Consequently, Cells on 200 nm nanochips displayed the least percentage of focal adhesions. In this case, 50 nm nanochips served as the transition inducing factor. Furthermore, the decrement in the percentage of focal adhesions as the nanochips became greater than 50 nm was consistent with the behavior of cells in the early stages of Serous (Fig. 5b) and Mucinous type of Ovarian cancer (Fig. 6). In the advanced stages (IVB), a continuous decrease in the percentage of focal adhesions with the increase of nanochips from 10 to 200 nm was observed (Fig. 7b). Consequently, 200 nm displayed the least percentage of focal adhesions. This was in firm consistency with the findings in the advanced stage (IVB) of the Serous type of Ovarian cancer (Fig. 5b).

The percentage of MFB in the early stage (IA) on 10 nm Nanochips was less as compared to flat surfaces. The percentage of MFB then increased from 10 nm till the nanochip size reached 100 nm and then decreased (Fig. 7c). 100 nm nanochips induced the transition in the decrement of MFB in this case. This trend was found to be consistent with the cells in the early stage (IA) of Serous type carcinoma cells (Fig. 5c). Overall, the decrease in the MFB for cells on 200 nm nanochips was consistent in early stages of all the three types (Serous, Mucinous, and Clear Cell) of cancers (Figs 5c, 6 and 7c). A similar trend of decrease in the percentage of MFB with the increase in the nanochip size was seen in stage IIIC. In addition, in the advanced stage (IVB), the MFB first increased on 10 nm till the size reached 50 nm and then decreased consistently (Fig. 7c). 50 nm nanochips induced the transition in the percentage of MFB. Cells on 200 nm nanochips displayed the least percentage of MFB. Interestingly, the trend was consistent as seen in the advanced stage (IVB) of Serous type carcinoma (Fig. 5c).

The cell area measurements complimented the morphology transition (Fig. 4) in different stages of the Clear cell carcinoma. In the early stages (IA), cells on 10 and 50 nm nanochips depicted a well-extended morphology (Fig. 4) which accounts for the large area of a cell (Fig. 7d). However, as the nanochips became greater than 50 nm, cell area values dropped (Fig. 7d) which was complimented by an elongated morphology (Fig. 4) as shown by cells on 100 and 200 nm nanochips. In the advanced stage (IVB), cell area decreased consistently with the increase in the nanochips’ size. This was in agreement with the morphology (Fig. 4) of the cells where cells started adopting a spindle-shaped morphology for the nanochip size greater than 10 nm. Therefore, nanochips greater than 10 nm induced the morphology transition in the advanced stage of clear cell carcinoma (Fig. 4). In all stages, cell area on 10 nm nanochips was consistently found to be less than the cell area on flat surfaces.

## Discussions

Cellular microenvironment plays a very important role in guiding the cells’ characteristics^40^,^41^. It has been verified that cellular microenvironment generates signals which guide the growth/differentiation of the surrounding cells^42^,^43^. Furthermore, as shown by some recent studies, the local cellular microenvironment also plays a crucial role in guiding the cells of Primary tumors^32^. The elements of the cellular microenvironment act as cues to guide the tumor progression^44^. This in turn also causes changes in the local cellular microenvironment. This implies that the cellular microenvironment and its components guide and regulate each other^45^. Since the components of the cellular microenvironment lie in the nano-range, it is possible that these components may be able to interact with the artificial nanoscale features. A plethora of studies have been done in the past to elucidate not only the nature of interactions between the cellular entities and the materials with surface features in nanoscale but also the changes induced by these nano-topographic features in the cells on the genetic level^1^,^3^,^24^. For instance, our research group in the past has performed multiple studies on materials such as stainless steel to study how cellular entities interact with their micro/nano environment by designing different topographies^5^,^26^. However, no study until now has aimed to fabricate a platform which can be employed to study not only the nature of interactions but also the modulation of cancer cell characteristics and identifying which nanodot size can induce a transition in the cell characteristics by using clinical cancer samples as a measure to investigate the invasiveness of cancer cells. In this study, we have fabricated nanochips of Tantalum oxide nanodot arrays to act as artificial microenvironments by mimicking the *in-vivo* tissue microenvironment. We used Clinical Ovarian cancer samples of different types and in different stages to investigate if our nanochips can modulate the cell characteristics differently in different stages of the cells. We fabricated 4 different nanochips of Tantalum Oxide nanodot arrays of different sizes (10, 50, 100 and 200 nm) and defined 4 different parameters (Cell Viability, Focal adhesions, microfilament bundles, Cell morphology/Cell area) to investigate their modulation as a measure to study the invasiveness of Ovarian cancer cells.

Our first task was to check if the nanochips successfully modulated the morphology in different stages of a given type of Ovarian cancer. Our results after seeding the cells for 3 days indicated that the nanochips of different sizes acted as different artificial microenvironments to induce a transition in the cell morphology in different stages of the Ovarian cancer cells (Figs 2, 3 and 4). Cells displayed a spherical morphology in nanochips of 10 to 100 nm in the early stages (Serous IA) which transitioned to an elongated morphology of cells seeded on 200 nm nanochips (Fig. 2). However, in the advanced stages, cells displayed a spindle-shaped morphology (Serous IIIC, IVB). In contrast, cells displayed an elongated morphology in the early stages of Clear Cell type (IA) (Fig. 4) which transitioned to a shrunken morphology stage IIIC and a spindle-shaped morphology in IVB. Our results are consistent with the previous studies conducted on the morphology of cancer cells in the *in-vivo* tissue microenvironment. Studies on breast cancer in the past have concluded that a spindle-shaped morphology signifies a highly invasive cancer form^46^. The mentioned study was conducted based on isolating cells from the cellular microenvironment. However, in our current study, similar findings of spindle-shaped morphology (Fig. 4) in advanced stages of cancer indicate that our nanochips have successfully acted as artificial microenvironments to modulate cell characteristics. One reason for the difference in the modulation of morphology is the different origin of the two cell lines (Serous and Clear Cell). This implies, that having known the type of cancer cell, these nanochips can be used to study/define the stage (invasiveness) of that type of cancer cell based on modulation of morphology by them (Figs 2, 3 and 4). In the next step, we investigated the modulation of cell characteristics by the nanochips (Figs 5, 6 and 7). Our results indicated that the nanochips of different sizes modulated the cell characteristics to a different extent. However, the trend of modulation was consistent between same stages of 2 different types of cancer. For instance, increase in the cell viability in the early stages (IA) of Serous (Fig. 5a), Mucinous (Fig. 6) and Clear cell (Fig. 7a) with the increase in the size of the nanochips and consistent decrement of Viability in the advanced stages (IVB) of Serous and Clear cell and Mucinous (IC). These modulations imply that the nanochips controlled the cell viability irrespective of the type of Ovarian cancer. Furthermore, the continuous decrement in the percentage of focal adhesions (Figs 5b, 6 and 7b) is not only consistent with the same stages in different types but also with our previous findings^5^. In addition, our research group has previously demonstrated the modulation in morphology, cell indices such as apoptosis, viability, focal adhesions by studying the response of TOV-112D, TOV-21G, ES2, NIH-3T3^23^.

The experimental set-up and methodology of the present study may be limited to Ovarian cancer, however, this gives us new opportunities in designing platforms to study the modulation of cell characteristics by the Tantalum oxide nanodot arrays as an artificial microenvironment to study the invasiveness of any other type of cancer. For instance, we have shown in our previous studies on cervical cancer cells (HELA, C-33 A) that nanotopography modulates the apoptosis, focal adhesions, microfilament bundles differently in different cell lines. Moreover, new materials such as TiO_2_, PDMS^47^,^48^, TCPS^49^ are also consistently used to study the nature and modulation of interactions between cells and its micro/nano environment as a function of the size of surface features^50^. Owing to its excellent X-Ray visibility and magnetic susceptibility, Tantalum has also been widely exploited in fabricating stents^51^. Quite interestingly, cells not only respond differently to different sized nano surface features but also to the different shapes and roughness of the nanomaterial as is reported by Chen *et al*.^52^. All of these studies collectively point towards the multiple factors which need to be considered before designing a generalized platform to study the cellular characteristics since nanotopography with size greater than 100 nm has been seen to induce apoptosis, as is shown in our previous findings^23^. Additionally, even though the results of this study are based on the modulation of 4 different parameters, more experiments need to be done to identify more such parameters which are modulated by a nanomaterial to fabricate a generalized platform which can be used universally to study the invasiveness of any kind of cancer. The identification of other parameters as well as the effect of other nanotopographic features such as the inter-nanodot distance on these nanochips will also remain to the work of the future studies.

According to the results of this study, nanochips comprising of Tantalum oxide nanodot arrays ranging in size from 10 to 200 nm (Fig. 1b) can successfully modulate the characteristics of the Ovarian cancer cells which can be as factors to study the Ovarian cancer the invasiveness of cancer cells irrespective of the type of the cancer type. The findings of the present study may help in designing marker-less diagnostic platforms to study cancer invasiveness, in designing the surface of implants and in the field of Biomedical engineering and cancer research.

## Conclusions

In the present study, we have engineered nanochips of Tantalum oxide nanodots arrays ranging from 10 to 200 nm in diameter (Fig. 1b). The nanochips successfully modulate morphology (Figs 2, 3 and 4) and cell characteristics (Figs 5, 6 and 7) of the cells in different stages and of different origin. The nanochips of different sizes induced transition to a different extent in different stages of Ovarian cancer. Cells adopted an elongated/round morphology in the early stages of Ovarian cancer (Figs 2 and 4) which transitioned to a spindle-shaped morphology in the advanced stages (Fig. 4). A greater percentage of viable cells were seen in the initial stages of Serous (Fig. 5a), Clear cell carcinoma (Fig. 7a) in nanochip size greater than 100 nm while in the advanced stages of both type of carcinoma, the viability dropped drastically. The percentage of focal adhesions in the early stages of Serous (Fig. 5b), Mucinous (Fig. 6) and Clear cell carcinoma (Fig. 7b) decreased consistently with the increase in the nanochip size, however, the advanced stages of Serous and Clear cell carcinoma displayed almost negligible focal adhesions on the nanochip size greater than 100 nm. The percentage of MFB was also seen to be modulated by the nanochips (Figs 5c, 6 and 7c). MFB decreased in both early and advanced stage of Serous (Fig. 5c) and Clear cell carcinoma (Fig. 7c), however, the decrease in the advanced stages was more drastic.

According to the results of this study, nanochips comprising of Tantalum oxide nanodot arrays of size ranging from 10 to 200 nm may be exploited to fabricate a platform for studying and distinguishing between the invasiveness of Ovarian cancer. Furthermore, the transition in the cell parameters by the nanochips of a specific size can also be implemented in designing implant surfaces. The results of this study may mark a cornerstone in the marker-less diagnosis of Ovarian cancer and may also be exploited in the fields of Biomedical engineering, drug development, and cancer research and treatment.

## Methods

### Chemicals

Glutaraldehyde and osmium tetroxide were purchased from Electron Microscopy Sciences (USA). Anti-vinculin mouse antibody was purchased from Abcam (USA). Alexa Fluor 594 phalloidin and Alexa Fluor 488 goat anti-mouse IgG were purchased from Invitrogen (USA). Hoechst 33342 nuclear stain was purscared from Invitrogen (Thermo Fisher Scientific Inc. USA). Bovine serum albumin (BSA) was purchased from GIBCO (Thermo Fisher Scientific Inc. USA). Phosphate buffered saline (PBS) was purchased from Bio-tech (Taipei, Taiwan). Sulfuric acid (H_2_SO_4_), oxalic acid (H_2_C_2_O_4_), and phosphoric acid (H_3_PO_4_) were purchased from Sigma Chemicals (Perth, Western Australia), 6-inch silicon wafers, Aluminum ingots were purchased from Admat-Midas (Norristown, PA, USA). Paraformaldehyde, Triton X-100 were purchased from Alfa Aesar (Taiwan). Other chemicals of analytical grade or higher were purchased from Sigma or Merck (USA).

### Development of nanochips comprising of Tantalum oxide nanodot arrays

A think film of Tantalum Nitride (TaN) of 200 nm thickness followed by a 400 nm thick layer of Aluminum was deposited on a 6-inch Silicon (Si) wafer. The wafers were cut into smaller pieces of 3 cm length and 3 cm width and anodization was then carried out. For 10 nm nanodot arrays, anodization was carried out in 1.8 M Sulphuric acid at 5 V. For fabrication of 50 and 100 nm nanodot arrays, 0.3 M Sulphuric acid was employed and anodization was carried out at 25 and 50 V respectively. For 200 nm nanodot arrays, 5% (w/v) Phosphoric acid was used and anodization was carried out at 100 V. Porous anodic alumina formed during this process was removed by immersing all nanodot arrays in Phosphoric acid overnight. The anodized portion of the wafer was then cut using a diamond cutter. TaN-coated Si wafers were referred as Flat nanochips. Substrates consisting of 10, 50, 100, 200 nm Tantalum oxide nanodots arrays were referred as 10, 50, 100 and 200 nm nanochips. Consequently, 4 nanochips with 4 different nanodots diameters were engineered to serve as different artificial microenvironments to the cells. Nanochips with nanodots diameter less than 10 nm could not be fabricated due to the inability of the experimental setup to generate homogeneous geometry below 10 nm.

### Surgically Dissected Tumor Samples

A total of 16 surgically resected tumor samples, confirmed to be adenocarcinoma by histopathological examinations, were obtained from consecutive patients with different type of Ovarian cancer. Written informed consents were obtained from the patients with the study protocol approved by the institutional review board (IRB) from China medical university hospital. The IRB numbers are DMR100-IRB-254 and CMUH104-REC2-075. Informed consent was taken from all the patients. The resection was carried out in accordance with the relevant guidelines of China medical university hospital.

### Isolation and culture of Ovarian cancer cells from tissue or ascites

Primary or secondary tumors were obtained at the time of surgery from 16 different patients as described above. Fragments of the tumor were rapidly transferred into a sterile Universal container containing RPMI 1640 and placed on ice to maintain the viability. The universal container was transferred to a sterile environment. Tumor fragments were mechanically dissociated using crossed scalpel blades. (Swann-Morton, Sheffield, England). The cell suspensions were then filtered through a cell strainer (BD Falcon) to remove all the clumps. The cells were then resuspended in tissue culture medium and cultured at 37 °C, 100% humidity and 5% CO2 in a tissue culture incubator. The fibroblasts attach to the plastic more rapidly than the epithelial cells and the cells that attach within the first 2–3 hours are predominantly fibroblasts. The cell suspension was then transferred to a new flask after this period. Many of the fibroblasts remained in the original flask, which were then discarded. Tissue culture medium was replaced 2 times/wk^53^. 20 ml of ascitic fluid was aliquoted into a T75 flask containing 20 ml growth medium (RPMI1640 supplemented with 10% FBS and 100 units/ml penicillin, 100 μg/ml streptomycin). Ovarian tumor cell clumps or grape-like clusters were apparent in the ascetic fluid, which eventually adhere to the cell culture plate surface. After 3–4 days remove supernatant, the clumps were washed with 1X PBS and fed with growth medium^54^.

### Immunostaining of Actin and Vinculin filaments

Cells were harvested after 3 days, followed by fixation with 4% Paraformaldehyde in PBS for 15 minutes. The samples were then washed 3 times with PBS followed by membrane permeabilization in 0.25% Triton X-100 for 10 minutes followed by 3 PBS washes. Non-specific binding was avoided by incubating the permeabilized cells in 2% Bovine Serum Albumin (BSA) for 1 hour at room temperature. The samples were then incubated in Rabbit anti-Human Vinculin antibodies (1:100, Diluted in 1% BSA) and Phalloidin for 1 hour, washed 3 times with PBS, followed by incubation in Goat anti-Rabbit FITC conjugated secondary antibodies (1:100, Diluted in 1% BSA) for 1.5 hours. Morphology and the number of focal adhesions (Green spots of vinculin) were analyzed with the help of Leica TCS SP2 confocal microscope. All washes were carried out using 1X concentration of PBS.

### Analysis of Cell Indices

Cell Indices namely Cell Viability, focal adhesions, microfilament bundles, cell Area were evaluated. To evaluate cell viability, cells were harvested after 3 days, the nucleus was stained WITH Hoechst 33342 nuclear stain (2 μg/ml) and the number of viable cells on different nanochips (Flat, 10, 50, 100, 200 nm) were counted. For analyzing the number of focal adhesions, the number of green spots (Vinculin) per cell were counted (shown as arrows on Fig. 2(a–c)). To analyze the microfilament bundles, a 3-D cell image was constructed using the confocal microscope and an estimate of bundles of microfilaments protruding from cells to the nano-surface was made. Cell area was analyzed by using Image J software package (NIH). For calculation of each cell index, six readings were taken from each nanochip (Flat, 10, 50, 100 and 200 nm) and the average was plotted against the different size of nanodot arrays (Flat, 10, 50, 100 and 200 nm). The measurements of all indices on all nanochips were normalized against the control surfaces (Flat Silicon). For measuring each cell index, six different substrate fields were measured per sample and three separate samples were measured for each nanochip.

### Statistical Analysis

The experiments were repeated three times on Nanochips. The data were expressed as the ± standard deviation. Statistical analysis was performed by using SPSS.

## Additional Information

**How to cite this article**: Dhawan, U. *et al*. Nanochips of Tantalum Oxide Nanodots as artificial-microenvironments for monitoring Ovarian cancer progressiveness. *Sci. Rep.* **6**, 31998; doi: 10.1038/srep31998 (2016).
